# Essential nucleic acid omics: a theoretical foundation for early-stage users

**DOI:** 10.3389/fbinf.2025.1721028

**Published:** 2026-02-04

**Authors:** Andrew J. Maritan, Frank J. Stewart

**Affiliations:** 1 Montana State University, Department of Microbiology & Cell Biology, Bozeman, MT, United States; 2 Max Planck Institute for Marine Microbiology, Bremen, Germany; 3 Georgia Institute of Technology, School of Biological Sciences, Center for Microbial Dynamics and Infection, Atlanta, GA, United States

**Keywords:** beginner, early career, FAIR, guide, ISA, MAGS, metabarcoding, pipeline

## Abstract

Modern biology often relies on the analysis of entire sets of molecules (omics). A subset of omics uses nucleic acid sequencing to reconstruct genomes and profile gene expression. Novel findings and existing data are contextualized by databases, which have been growing exponentially due to falling sequencing costs and increased computing access. The increasing accessibility of omics has led to rapid adoption and widespread self-training via open-access tools. In this training environment new users (many of whom are students also applying computing for the first time) are confronted with Terabytes of sequence data and an ocean of topic-specific computing guides (often directed at high-level users). This flood of information creates an initial barrier of confusion and frustration, where it is challenging to identify the overarching goals of omics analyses through the details of computing. We believe this confusion is understandable but not pre-destined, as omics is–at its core–simple. This simplicity comes from its modular nature, where any analysis requires familiarity with only a few consistent steps. Here, we identify core elements of all omics analyses–data products, tools, and workflows–using microbiology applications to ground the discussion. This structure is informed by first-hand experience training early-stage omics users, where covering omics theory provides a foundation for practical implementation.

## 1 Introduction

Analyzing nucleic acid sequences (omics) is a universal tool in contemporary biology. In this world, new biologists benefit from understanding the foundational motivations and methods of omics analyses whether or not they intend to apply these tools themselves. In our experience teaching omics to students for both future use and background context, we see that most are competent biologists lacking computing experience. For these students, the technical details of computing often obscure the fact that omics analyses are simple arrangements of a few modular tools, producing a few consistent outputs. To highlight the simplicity of omics, we focused this review on broadly applicable theory to provide a view of the omics “bigger picture”. Further, to avoid distracting from this perspective, we limited our discussion of the technical details of computing, which is available in other excellent guides as needed (see “Section 5.1.1 Opportunities for Further Training”).

We organized this review in four parts, providing an increasingly granular understanding of nucleic acid omics. Part 1 (Section 2) describes the biological goals of different types of omics by discussing the history of their development. Part 2 (Section 3) identifies the analytical goals of omics by identifying the core data products. Part 3 (Section 4) describes the repeated modular steps of omics analyses that are used to generate core data products. Part 4 (Section 5) concludes with computational and non-computational tips for new users. The review’s structure ensures that a student can read it completely for a top-to-bottom guide to nucleic acid omics, or individual sections to clarify specific questions. For some readers, this review will be sufficient to understand the “methods” sections of manuscripts, while others will want to continue with more specific training to run omics analyses independently. For both groups, this review should make nucleic acid omics more tractable, providing a foundation for engaging more deeply with omics literature and/or code.

## 2 The development of omics: a short history

### 2.1 What is omics?

The term omics describes analyzing a system (single cells, organs, organisms, or communities of organisms) using its biological molecules (DNA, RNA, proteins, metabolites). The biological molecule in-question determines the name of the omics analysis (DNA: gen-omics, RNA: transcript-omics, proteins: prote-omics, metabolites: metabol-omics), while the system’s scope determines the prefix (“meta-” applies to community studies: meta-genomics, meta-transcriptomics, while no prefix is applied to single-species studies). In this review, we will focus on nucleic acid-based omics techniques, using the terms “genomics” and “transcriptomics” for consistency, though the topics are applicable to community scale meta-omics (Box 1). Nucleic acid sequences are the foundation of omics as they (especially genomes) provide the near-complete repertoire of life’s function-encoding units (protein coding genes and transcripts, rRNA, tRNA, …). Many of these coding units are conserved across the domains of life allowing researchers to assign likely function and taxonomic identity to newly acquired sequences by comparing them to ever-expanding reference databases. These modern technical capacities were developed over decades, and understanding this history–especially past limitations–is essential for adding new data into a historic literature (Brock, 1999). To that aim, we will cover a brief history of the development of modern nucleic acid omics.

#### 2.1.1 Nucleic acids and the central dogma

The groundwork for modern omics was laid by identifying DNA as the molecule of genetic inheritance (Avery et al., 1944), description of DNA’s structure (Watson and Crick, 1953), and the articulation (Crick, 1958) and experimental support (Gros et al., 1961; Brenner et al., 1961) of the Central Dogma of molecular biology: genetic information stored in DNA, transmitted by RNA, and manifested as proteins. This biochemical linkage means that the study of any of these molecules informs the understanding of their precursors or derivatives.

#### 2.1.2 Marker genes

Informed by the Central Dogma, particular gene sequences (DNA and RNA) proved to be especially predictive (e.g., phenotype, evolution, heredity, behavior) and are termed “marker genes” (Box 1). Study of marker gene distribution and variance to understand biological phenomena was the direct precursor to omics approaches (marker gene analyses are not always considered “omics” because they do not capture “entire subsets of molecules”, though we discuss marker genes throughout this review as they are related to other DNA and RNA-based approaches).

**FIGURE 1:**
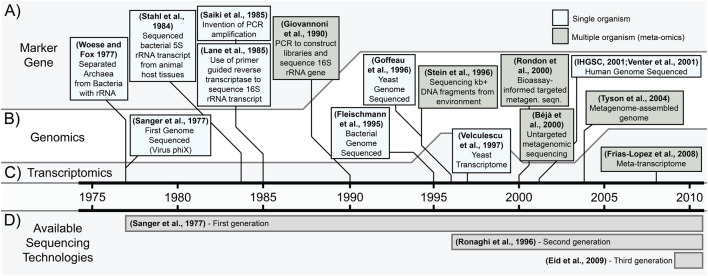
Illustrative timeline of the development of nucleic acid omics. Included are the first examples of technologies that enabled–or are–modern omics between 1975 and 2010, covering **(A)** marker gene sequencing, **(B)** genomics, **(C)** transcriptomics, and **(D)** the availability of different sequencing technologies–shaded from their announcement to the end of the timeline. IHGSC = International Human Genome Sequencing Consortium.

A landmark example of marker gene analyses used the conserved and abundant ribosomal RNA (rRNA; Figure 1A) to study evolution across the tree of life. Using rRNA digestion fragmentation patterns on gels, Archaea were discovered in 1977, upending conceptions of the origin of Eukaryotes (Woese and Fox, 1977). Purified rRNA remained a popular molecule for reconstructing phylogenies, with methods developed to sequence it directly (Stahl et al., 1984; Lane et al., 1985; Figure 1A; Box 1). In parallel, methods developed to multiply and sequence the less abundant DNA fraction, opening the possibility of examining other marker genes, though rRNA remained a popular target to study evolution. Initial sequencing of DNA marker genes required cloning target genes into viral vectors–notably used to place the bacterial origin of the mitochondrion (Yang et al., 1985) – a labor and resource intensive effort. Acquiring enough DNA to sequence marker genes was greatly simplified by the invention of PCR in 1985 (Saiki et al., 1985), allowing near-direct sequencing of low abundance DNA encoded genes. This method was soon applied to study rRNA genes in mixed microbial communities in 1990 revealing previously unknown diversity (Giovannoni et al., 1990).

Beyond the use of rRNA to assign taxonomy and reconstruct evolutionary lineages, marker genes can also be used to screen organisms for pre-selected functions ranging from diagnosing sickle-cell anemia in humans (Kan and Dozy, 1978) to the identification of nitrogen fixing bacteria in the ocean (Tschitschko et al., 2024).

Marker gene analyses upended evolutionary biology, radically increased our catalogue of biodiversity, and made it possible to understand phenotypes without direct observation. Marker gene analyses continue to be useful in studying genes already identified as important, though they only provide “snapshots of organisms” (Pace, 1997). Organisms are constructed from hundreds to thousands of genes (Hou and Lin, 2009), and a single marker gene only explains a tiny percentage of any organism’s genetic potential. Genome sequencing is required to understand co-occurring genes in a single organism and is the domain of genomics.

#### 2.1.3 Genomics

Genomics (Box 1) provides greater coding context than single marker genes and emerged with the publication of the first genome in 1977 (the virus phiX; Sanger et al., 1977a; Box 1) and ushered in the age of analyzing large collections of genes (Figure 1B). This first viral genome was small (5,386 bases) but was followed up with genomes from bacteria (*Haemophilus influenzae*, ∼1.83 Mbp; Fleischmann et al., 1995) and eventually humans (∼6.3 Gbp, diploid; (International Human Genome Sequencing Consortium et al., 2001; Venter et al., 2001; Figure 1B). Larger genomes were increasingly complex, but all possessed the conceptual simplicity that all sequences originated from a single organism. The concepts of single organism sequencing were quickly applied to sequencing genomic material from complex microbial communities. Multi-species microbiomes were sequenced by first inserting large (50+ kbp) fragments of DNA into *Escherichia coli* and later sequencing these clone libraries either randomly to survey the community (Béjà et al., 2000), or deliberately to capture specific taxa (Stein et al., 1996) or functions (Rondon et al., 2000). This sequencing of genomic DNA from multi-species communities were the first metagenomes, expanding the knowledge of protein-coding gene diversity and the environmental distribution of metabolic functions and taxa (DeLong et al., 2006; Yooseph et al., 2007). Further, genes encoding taxonomy and function often co-occurred on a single large fragment (Stein et al., 1996; Béjà et al., 2000) allowing researchers to describe an organism solely through molecular data–first identifying it (see marker gene above) and then hypothesizing its “functional potential”. This work linking taxonomy-to-function in metagenomes advanced when metagenomic sequences were used to reconstruct individual complete (or near-complete) microbial genomes (Tyson et al., 2004). These metagenome-assembled genome (MAG; Parks et al., 2017; Yutin et al., 2021; Box 1) and non-MAG (Dragone et al., 2022; Bertagnolli et al., 2023) approaches are now widely applied on diverse uncultured microorganisms to understand their taxonomies and functional potential. The distinction of “functional potential” is essential, as genomes provide evidence that a function might be performed, but do not demonstrate activity (Hatzenpichler et al., 2020). Activity can be more accurately approximated by studying gene expression–a sort of “metabolic intention” – which is the domain of transcriptomics (Box 1).

#### 2.1.4 Transcriptomics

To understand “metabolic intention” the sequencing methods of DNA pools were applied to RNA (following reverse transcription of RNA to cDNA), creating the field of transcriptomics. Early sequencing of untargeted RNA provided initial insights into the diversity of expressed genes in different cell types, requiring the cloning of individual cDNA transcripts into *E. coli* clones (Adams et al., 1991). Accurately quantifying RNA expression–allowing rigorous comparison between cell types–became possible with the advent of microarrays. There, cDNA from thousands of pre-selected gene targets were attached to glass slides and then hybridized with experimentally sourced (extracted and reverse transcribed) cDNA, creating fluorescence proportional to the sample cDNA, allowing quantification (Schena et al., 1995). Derivatives of these technologies are still in-use today and set the stage for “transcriptomics” where RNA sequencing was used in-concert with existing genomes to identify expressed genomic regions (Velculescu et al., 1997), which began using “Serial Analysis of Gene Expression” (SAGE). In SAGE-based transcriptomics, cDNA was sequenced by cleaving each cDNA transcript into a short tag (9–11 bp), the tags concatenated into a longer sequence, cloned into *E. coli*, PCR amplified, and sequenced. These tags were then extracted bioinformatically, aligned against a reference genome, where alignment (Box 1) of an RNA tag to a DNA sequence indicated gene expression and the number of tags aligning to any DNA sequence used to quantify expression (Velculescu et al., 1995; Velculescu et al., 1997). SAGE transcriptomics was first used in 1997 on yeast cultures with RNA tags aligned to the new yeast genome (Goffeau et al., 1996) to generate maps of thousands of expressed genes (Velculescu et al., 1997; Figure 1C). Advances in sequencing (RNA-seq; Bainbridge et al., 2006; Nagalakshmi et al., 2008), resulted in more and longer RNA sequences (beyond SAGE’s 10s of bp tag approach) increasing its sensitivity (identifying splicing and lowly expressed genes), read coverage, and data volume. As transcriptomics advanced, it was applied to mixed-species microbiomes, revealing gene expression by dominant (Frias-Lopez et al., 2008; Hewson et al., 2010; Stewart et al., 2012) and less abundant taxa (Stewart et al., 2012) in the environment.

#### 2.1.5 Perspectives

In the last century, biologists have learned that DNA is the molecule of trait inheritance and can now measure gene expression from nanogram quantities of RNA in the wild. These new approaches have enabled sequence-based investigations of diversity and function across earth (Sunagawa et al., 2020; Nayfach et al., 2021; Shaffer et al., 2022) and into space (International Space Station; Castro-Wallace et al., 2017), surpassing the dreams of early omics scientists (Pace, 1997).

These global surveys of commonplace (seawater, soils, human skin) and extreme (hot springs, alkaline lakes) environments were essential to fill the complete vacuum of information about the diversity and distribution of uncultured microorganisms. However, the number of unexplored ecosystems shrinks daily, and as-such, modern microbiologists should not expect that sequence-based surveys will provide the acclaim of the early days of sequencing.

Today’s omics researchers should follow the example of early scientists to answer specific biological questions (reconstructing the tree of life, Woese and Fox, 1977; reconstructing the evolution of symbionts; Lane et al., 1985; Yang et al., 1985) using available tools. The basic toolkit of omics is well established (at least since 2008; Figure 1), but advances in rapid cheap sequencing and ∼50 years of archived sequencing data have produced opportunities to answer new biological questions with global samples (The Earth Microbiome Project, Thompson et al., 2017; TARA Oceans; Sunagawa et al., 2020) and replication across space and time (the National Science Foundation’s: National Ecological Observatory Network, Dantzer et al., 2023; and Long Term Ecological Research Network; Knapp et al., 2012). Zooming-in, omics now has the capacity to sequence the genomes (Raghunathan et al., 2005; Woyke et al., 2009) and transcriptomes (Ma et al., 2023) of single cells; part of a broader interest in understanding heterogeneity between single cells (Hatzenpichler et al., 2020; Kitzinger et al., 2020; Marlow et al., 2020). Microbiology’s newfound acquisition of spatially resolved (micron to global) and longitudinal (decades) sequencing data is one exciting new frontier for omics research (Eren and Banfield, 2024).

### 2.2 Nucleic acid sequence analyses are everywhere

Omics use has grown exponentially since its inception (Gauthier et al., 2019). One indicator of omics use is the rate of sequence deposits into reference databases. The NCBI Sequence Read Archive (the major public repository for unprocessed sequence data globally) has added 25.6 Petabase pairs (2.56 × 10^16^ base pairs - the data equivalent of ∼6,500,000 human genomes; Nurk et al., 2022) from 2012 to 2021 (Katz et al., 2022). The NCBI GenBank (a repository for assembled sequence data) has doubled in size every ∼2 years from 2013 to 2024, to a total of 3.4 × 10^13^ bp (Sayers et al., 2025). The number of available reference genomes also indicate use, with the number of human genomes doubling every 7 months from 2001 to 2015 (Stephens et al., 2015). This exponential data production has been accompanied with a proportionate development of new bioinformatics approaches (Gauthier et al., 2019), with a conservative estimate of 25,000 unique bioinformatics tools produced between 1990 and 2017 (Clément et al., 2018). This flood of data and tools has created an application bottleneck, where many omics practitioners simplify analytical decisions by focusing on the straightforward aim of recovering genomes to describe the metabolism of focal taxa. This simplifying approach makes sense in-light of abundant data and tool options, but we believe that reducing complexity through genome-only analyses is unnecessary. The apparent complexity of nucleic acid omics is illusory, with all omics analyses built on a simple and consistent set of data products and methodologies. In this review we will distill diverse omics analyses–extending beyond genomes–into their shared data products, the classes of tools to generate them, and how these tools and data are strung together into workflows to answer biological questions. We will begin by describing the five core omics data products.

## 3 Omics data products: a few goals

**FIGURE 2:**
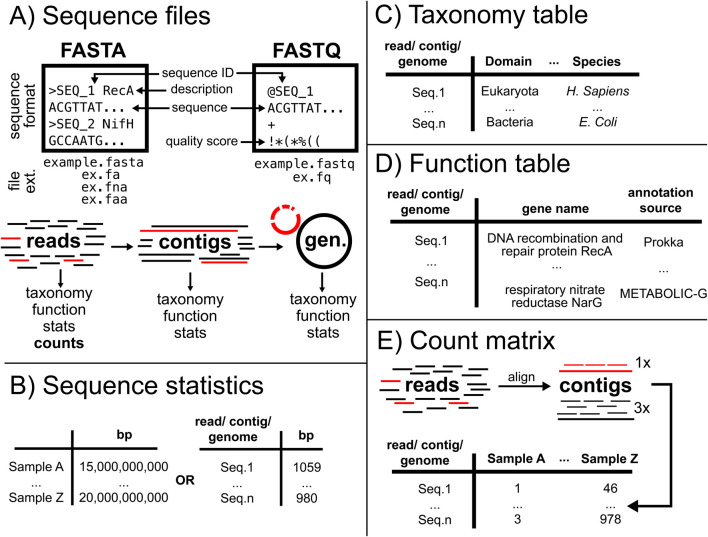
All nucleic acid sequence-based omics analyses rely on five core data products. Simplified examples of the core data products. **(A)** Sequence files (two common formats depicted) are used to encode reads, contigs, and genomes. They contain nucleic acid bases and some supporting metadata. Sequence files are used to derive all other data products. **(B)** Sequence statistics are used to describe the contents of a sequence file (often total number of sequences/base pairs or sequence lengths). **(C)** Taxonomy tables link sequences to likely source taxa. **(D)** Function tables link sequences to their likely functions. **(E)** Count tables are generated using read data and contain the estimated abundances of each sequence. Bp = base pairs; cond. = condition; ex. = example; ext. = extension; gen. = genome; seq. = sequence; stats = statistics.

Assuming the omics researcher has formulated a scientifically meaningful guiding question, the next step is to identify tractable computation goals: “Are we surveying functional and/or taxonomic content?”, “Do we need to contextualize these data phylogenetically?”, “Do we want genomes?”, “Do we need to quantify or statistically test our findings?”, etc. These procedural endpoints allow a bioinformatician to work backwards to construct an analytical workflow, identifying midpoint questions and target data products. These data products of omics (here: genomic, transcriptomic, and marker gene) fall into one of five classes: 1) sequence files, 2) sequence statistics, 3) taxonomy tables, 4) function tables, and 5) count tables. These data products are necessary to any omics analysis and must be incorporated into a larger biological narrative to be useful. With that in mind, we describe the general structure and uses for each of these data classes (summarized in Figure 2).

### 3.1 Sequence files

Digitized biological sequences are the foundation of all omics (sequencing described below; “Section 4.1.1 Sequencing Technologies”) and commonly follow the FASTA or FASTQ formats. The FASTA format contains both a sequence identifier and sequence data (nucleic acid residues; Figure 2A) while FASTQ contains the same information as FASTA files as well as quality scores for each nucleic acid residue (Q-scores). These quality scores allow the user to remove low-confidence sequences and/or bases (which then produces quality filtered FASTA files) before further analysis (Figure 2A). There are three major types of sequence files: 1) reads, 2) assembled contiguous sequences (contigs; Box 1), and 3) genomes. Each of these classes are used to generate the next (reads are used to make contigs, reads and contigs to make genomes) resulting in increasingly long context-rich sequences. We will now describe the characteristics of each of these sequence classes.

#### 3.1.1 Reads

Reads are the raw product of the sequencing platform and the basis of marker gene (16S rRNA gene), genomic, and transcriptomic studies. Reads are classified as “short” or “long” depending on the sequencing technology used to generate them and length of output reads. Short reads are tens to hundreds of bases long, while it is possible for long reads to be thousands to millions of bases (Satam et al., 2023). These reads contain all the (relative) abundance information in a sequence library (Gloor et al., 2017; Box 1), with derivative sequences (contigs and genomes) requiring reads for quantification.

Reads are the functional unit for marker gene studies, using fragments to full-length genes to identify microbial taxa (16S rRNA, *rpoB*; Thompson et al., 2017) or putative functions (*pmoA*, *narG*; Yu et al., 2024). In genomics and transcriptomics, read data are generally treated as a steppingstone to assembling contigs and recovering genomes. However, analysis of unassembled reads can be valuable as it uses the maximum amount of available data and therefore provides a relatively unbiased representation of microbiome gene content (Hauptfeld et al., 2024). Assuming individual reads are of a length sufficient for confident identification of homologous sequences (homologs) in a reference database, unassembled read datasets can be searched to identify taxonomically (Meier et al., 2017; Dragone et al., 2022) and functionally (Ortiz et al., 2021; Täumer et al., 2022; Maritan et al., 2025) informative marker genes. Read-based approaches can also be used to sift through reference databases to identify only the datasets that include metabolisms or taxa of interest (Speth and Orphan, 2018). Though read-based analyses have (often untapped) potential, the most common use of read data is the reconstruction of contigs, which we discuss next.

#### 3.1.2 Contigs

Contigs are generated by assembling reads into longer nucleic acid sequences. This approach is used in genomics to create genomic scaffolds (Prjibelski et al., 2020), and assembly-based transcriptomics to generate transcripts (Grabherr et al., 2011). Assembled contigs are, by definition, a subset of total sequencing effort, as not all reads can be placed into a contig (Hauptfeld et al., 2024). Despite data loss in assembly, contig sequences are useful for community taxonomic and functional reconstruction because their length enables more accurate identification of homologs compared to reads. If an assembled contig contains multiple protein coding sequences (genes of the same operon), this ‘genomic neighborhood’ (Wei et al., 2024) can be used to increase confidence in assigning gene function (Mihelčić et al., 2019) or taxonomy (Mirdita et al., 2021). Contig-derived genes are also potential inputs for phylogenetic reconstruction, enabling contig-based evolutionary and taxonomic analysis of a microbiome. Though analysis of standalone contigs is useful, the most common use of contigs is to reconstruct genomes.

#### 3.1.3 Genomes

Genomes are made by grouping (i.e., binning) contigs with similar features (details discussed below) into a single sequence file. In this review, we use the term “genome” to discuss a collection of sequences that likely come from the same organism, encompassing genomes recovered from pure cultures and mixed species consortia (termed: metagenome-assembled genomes; MAGs). Genome binning, like contig assembly, results in data loss (Hauptfeld et al., 2024) only examining a subset of the total microbiome. Despite this, genome-based analyses are appealing because they create a meaningful association among contigs, by which taxonomy or functional potential assigned to any contigs is passed onto all other contigs in the genome. This analysis allows a researcher to characterize the metabolic potential of individual microbes (Kohtz et al., 2024) and communities (Shoemaker et al., 2024; Ricci et al., 2025), even if the organisms containing these genomes have never previously been observed (Evans et al., 2015; Wurch et al., 2016). Genomes can also be used as references for aligning transcriptome sequences recovered from the same environment, thereby identifying expression patterns in individuals or communities across environmental gradients (Kitzinger et al., 2020; Porras et al., 2024). Beyond community description and reconstruction, genomes (Tripp et al., 2008) and transcriptomes (Bomar et al., 2011) can also be used to optimize cell culture (Wurch et al., 2016). The methods for generating and processing each of these types of sequence data are discussed in greater detail below.

### 3.2 Sequence statistics

Sequence statistics are derived from sequence files and have two major purposes: 1) contextualizing a narrative (describing dataset size/complexity, sampling effort, and/or similarity between sequences) and 2) normalizing count data. Viewing and analyzing these statistics typically involves generating tables of total bases in each sequence library or of individual sequences (reads, contigs, or genomes; Figure 2B). The methods for generating sequence statistics are discussed in detail below (“Section 4.2 Sequence Statistics”).

### 3.3 Taxonomy tables

Taxonomic classification (Box 1) aims to generate tables that relate a sequence identifier to a taxonomic lineage (Figure 2C). Taxonomic lineage is assigned to sequence data (reads, contigs, or genomes) by comparing unknown query sequences against reference sequences with known taxonomic origin. If the query sequence is sufficiently similar to a reference, the query is assigned the taxonomy of the reference (Goris et al., 2007; Jain et al., 2018; Parks et al., 2020). The methods for generating taxonomy tables are discussed in detail below (“Section 4.8 Taxonomic Classification”).

### 3.4 Function tables

Function annotation aims to generate tables that relate a sequence identifier to a descriptor of putative cellular function, often relating to metabolism, physiology, or behavior (Figure 2D; Box 1). Functional annotation of sequence data (reads, contigs, or genomes) compares an unknown query sequence against annotated (and potentially experimentally validated, although this is not always possible) reference sequences. There are at least two important caveats regarding using and interpreting functional annotations. First, the quality of any annotation is tied to the completeness and annotation accuracy of the reference database. Sequences from well represented model organisms (*E. coli*, *Pseudomonas* sp., etc.) and their close relatives can typically be annotated with high confidence, while genes in non-model organisms will be less confidently annotated (Goodacre et al., 2014). Second, while functions identified in genomic data indicate metabolic potential and functions identified in transcriptomic data indicate gene expression, neither genomic nor transcriptomic evidence of putative function proves that amino acids were translated or their proteins were active. The methods for generating function tables are discussed in detail below (“Section 4.9 Function Annotation”).

### 3.5 Count matrices

Sequence quantification (reads, contigs, or genomes) aims to generate tables that relate a sequence identifier to an estimate of its relative abundance in a sample, thereby providing a loose indication of a gene or organism’s biological significance (Figure 2E). Read quantification often involves simple counting, while quantifying longer sequences (contigs and genomes) requires aligning the source reads to the longer sequences (Aroney et al., 2025). Counts can be used as a descriptor of community composition (Bollati et al., 2024), to test hypotheses of differences in abundance of functional potential or taxa (Maritan et al., 2025), to identify associations between taxa and environment (Mitchell et al., 2024), or as input for quantitative modeling (Louca et al., 2016). The methods for generating count matrices are discussed in detail below (“Section 4.10 Count Data”).

### 3.6 Putting it all together: merging and using omics data

These data products are often the midpoint and endpoint goals of an omics workflow. Once data tables are generated (if all samples, metadata, and sequences have consistent naming), they can be merged into a “master table” for downstream filtering, plotting, phylogenetic inference, statistical tests, or other direct comparisons. However, merging tables without a specific goal may not be useful as it can create unwieldy tables with millions of columns or rows.

By clearly describing the core data products of all omics, we hope to make the endeavor less abstract. Thus far we have covered *what* is generated from omics (the five core data products), below we address *how* omics is executed via specific tools and workflows.

## 4 The omics toolkit: descriptions of common approaches, their purposes, and connections

**FIGURE 3:**
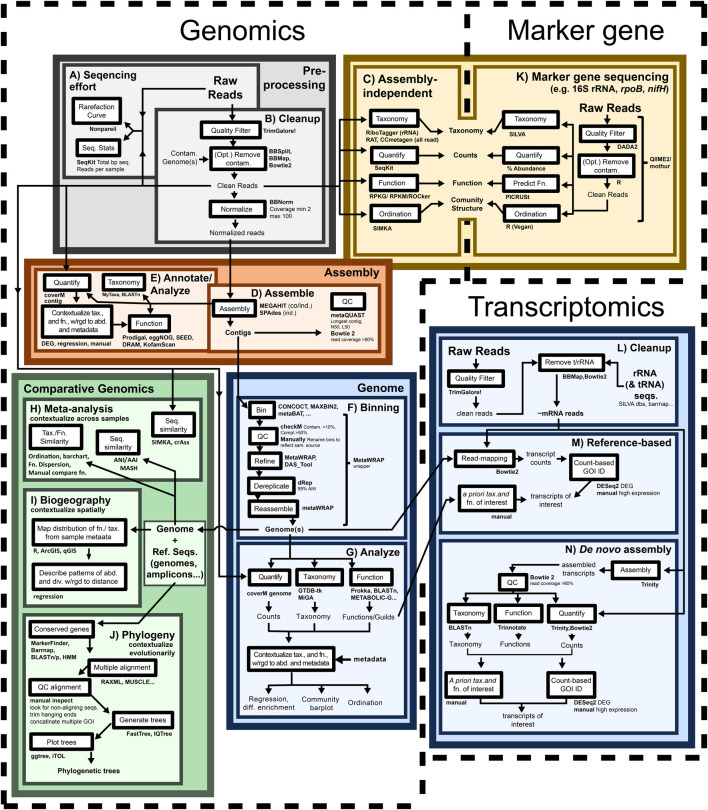
Nucleic acid sequence-based omics analyses are modular and complementary. Generalized workflow for performing marker gene, genomics, and transcriptomics analyses, with each demarcated by dashed lines. Within a single approach major processing steps are grouped within colored boxes, with inset, lighter boxes indicating subsidiary tasks. Discrete processing steps are named on white boxes (with example tools for performing the task alongside), colored boxes are used for clarity, but do not indicate importance. Data inputs and outputs are connected to processing steps with arrows. All pipelines begin with “Raw Reads” at the top of their respective approach. Marker gene: Marker gene analyses (from targeted amplification and untargeted genomics/transcriptomics) begin with quality filtering **(B,K)** and are then used to immediately generate counts and predict taxonomy and function **(C,K)**. Genomics: All genomic analyses generally begin by quantifying sequencing effort and calculating read statistics **(A)** and quality filtering reads **(B)**. Contig-based analyses assemble reads into contigs to generate count, taxonomy, and function tables **(D)**. Genome-based analyses use these contigs to generate genomes for subsequent generation of count, taxonomy, and function tables **(F,G)**. All genomic analyses are well suited for comparison against existing sequence databases **(H-J)**. Transcriptomics: Transcriptomic analyses begin by quality filtering reads **(L)**. There is then a split where some transcriptomics use genomics as a reference (reference-based; **(M)**) while others proceed independently and assemble RNA contigs (transcripts) requiring functional annotation and taxonomic classification of each transcript (*de novo* assembly; **(N)**) Both reference-based and *de novo* transcriptomics quantify expression by aligning RNA reads against the longer reference (genome or assembled transcripts) with the transcript aligned to determining taxonomy and function, with the number of reads aligning indicating expression (count table; **(G,N)**) Abd. = abundance, asse. = assembly; compl. = completion; contam. = contamination; db = database; DEG = differentially expressed genes; diff. = differential; div. = diversity; fn. = function; GOI = gene of interest; ind. = individual; opt. = optional; QC = quality control; ref. = reference; sam. = sample; seq(s). = sequence(s); tax. = taxon/taxonomy; w/wgd. = with regard to.

The toolkit of nucleic acid omics involves extraction and sequencing of nucleic acids with subsequent processing of generated sequences to make the data products outlined in Section 3. We now explore the methods available to do this, with each major computational step summarized in Figure 3.

### 4.1 Acquiring sequences

Sequencing is the basis of omics analyses with sequences generated *de novo* or downloaded from public databases. In either case, the quality and utility of any sequence dataset is underpinned by the quantity and length of output reads and confidence in the constituent bases–more and longer reads, with high confidence bases are markers of quality. These qualities are largely determined by the choice of nucleic acid extraction and sequencing technology.

#### 4.1.1 Sequencing technologies

Nucleic acid sequencing has had three major technological generations, each of which are still in-use and have pros and cons (reviewed in; Cheng et al., 2023; Satam et al., 2023). First generation sequencing is often referred to as “sequencing by termination” or “Sanger sequencing” after Frederick Sanger, its inventor and publisher of the first genome (Sanger et al., 1977a; 1977b; Figure 1D). This technology sequences one DNA molecule at a time, producing long sequences with low error rates (Cheng et al., 2023), and was used to achieve other genome “firsts” (bacterial, Fleischmann et al., 1995; yeast; Goffeau et al., 1996; human; International Human Genome Sequencing Consortium et al., 2001; Venter et al., 2001; Figure 1B). Today, Sanger sequencing is still in wide use: cheaply characterizing PCR amplicons from pure cultures and cloned genes, or in sequencing across gaps between contigs in draft genome assemblies (Drevinek et al., 2023; Katara et al., 2024). However, it is inefficient for processing dozens to hundreds of samples simultaneously (Panahi et al., 2024) – a need for efficient microbiome surveys–solved by later generations of sequencing.

Second generation sequencing is also known as “next-generation” or “short-read” sequencing” (Figure 1D) and is largely synonymous with the most prominent producer of short-read sequencers: “Illumina” (though expiring patents and new competitors are driving innovation and price reductions; Eisenstein, 2023; De Ronne et al., 2025). Short read sequencing generally entails spatially separating DNA fragments and observing the synthesis of bases (via fluorescence or pH change), producing short (25–300 bp) reads (Cheng et al., 2023; Satam et al., 2023). These reads can be analyzed with minimal processing (16S rRNA marker gene sequencing and transcriptomics) or assembled into contigs and binned into genomes. Genomes can be challenging to complete using short read data, because complex genomic regions are often longer than the technology’s maximum read length (∼500 bp; Satam et al., 2023; Panahi et al., 2024), preventing their reconstruction (Mise and Iwasaki, 2022). The read length limitation has been addressed by third generation sequencing.

Third generation sequencing is also known as “long-read sequencing” or by the trade names of the most prominent producers of long-read sequencers: Oxford Nanopore Technologies (ONT) or Pacific Biosciences (PacBio; Figure 1D). As the name suggests, long-read sequencing generates longer reads than second generation (10 kb+; Satam et al., 2023) which allows each read to capture greater genomic context (e.g., full length 16S rRNA genes, near-complete genomes). This technology passes (near-) full length nucleic acid molecules through a fixed sequencing unit (conductive pore or modified DNA polymerase), recording bases as they pass through. This technology can have higher error rates than short-read sequencing (Panahi et al., 2024), though it is possible to combine the higher quality short reads and the greater genomic context of long-reads to create long, high quality contigs (Antipov et al., 2016; see “Section 4.3.3 Contigs” below). Additionally, it is important to note that the name “long-read sequencing” indicates only a technological capacity–not a guarantee–to produce long reads. Sequenced read length depends on the length of the nucleic acids provided to the sequencer, which in-turn depends on minimally fragmenting nucleic acids during extraction, which we will discuss below.

#### 4.1.2 Nucleic acid extraction and sequencing

Generating new sequence data proceeds via two steps: 1) nucleic acid extraction and 2) sequencing, where the intended sequencing technology should inform extraction method. All nucleic acid extractions aim to lyse cells, expose nucleic acids, remove non-nucleic acid lysate, and collect enough nucleic acids to sequence anything. Though all these steps are important, the method of initial lysis largely determines sequencer compatibility.

Long-read sequencing requires minimally fragmented (high molecular weight) nucleic acids to produce long reads, whereas short read sequencing is less sensitive to fragmentation (Table 1; Zhang et al., 2022). For this reason, extraction for long-read sequencing should use “gentler” chemical lysis (detergents: SDS, solvents: Phenol-chloroform, TRIzol, or enzymes: lysozyme; Trigodet et al., 2022), while short read sequencing can combine chemical and mechanical (bead beating, freeze-thaw; Hamilton et al., 2011) lysis to maximize nucleic acid yields.

**TABLE 1:** Summary characteristics of short read and long read sequencers. Highlighting differences in read lengths and nucleic acid extraction methods.

Features	Short read (“second generation”)	Long read (“third generation”)
Usual Read Lengths (bp)	100s	10,000–1,000,000s
Common Manufacturers	Illumina, Element Biosciences	Oxford Nanopore, Pacific Biosciences
Optimal Nucleic Acid Extraction Methods	Mechanical (bead beating, freeze thaw) and/or Chemical (detergents, enzymes)	Chemical (detergents, enzymes)

#### 4.1.3 Data mining

Sample collection, extraction, and sequencing are all costly and can be reduced by using publicly available sequence datasets (NCBI SRA, Leinonen et al., 2011; NCBI nt/nr Sayers et al., 2024; EMBL UniProt; The UniProt Consortium et al., 2025). These datasets, combined with robust questions can be impactful (see “The Parasite Awards”, awarded “for rigorous secondary analysis of data”; https://researchparasite.com/). As examples, Kumagai et al. (2018) leveraged both public and newly sequenced genomes to explain the distribution of light harvesting proteins in marine bacteria. While Henriques et al. (2024) used publicly available vertebrate genomes to reconstruct the evolutionary trajectories of endogenous viral genes domesticated for host function in placental mammals. Though studies based on data mining are useful, it should be noted that papers centered around data mining are always limited by available resources. It should be noted that the use of others’ data requires careful attribution of the datasets used (citations, accession numbers) and potentially the consent of those who generated the data. Best practices for using and sharing public data should always be followed and are described in publishing policies in academic journals, or in review papers (Sielemann et al., 2020; Hug et al., 2025).

#### 4.1.4 Sequencing effort

For both new and mined read data, it is essential to consider sequencing effort. When sequencing genome(s), it is essential to sequence enough to capture the sequence diversity present in a sample. The relationship between sequencing effort and new information obtained follows a logarithmic relationship, where more sequencing recovers more and more novelty, until enough sequencing has been performed and novelty saturates. Identifying where a sample lies on the sequencing effort-to-novelty plot is a measure of sequence “coverage” which describes the fraction of the genome(s) represented by sequenced reads (Rodriguez-R and Konstantinidis, 2014). The number of reads needed to achieve high (>90%) coverage varies by system (Rodriguez-R et al., 2018), with larger genomes (human) and diverse microbiomes (sediments) requiring more sequencing effort than small genomes (phiX) or simple microbiomes (hot springs). A sample with suboptimal coverage can still be analyzed, but with the caveat that the analysis will be incomplete due to unidentified sequences.

#### 4.1.5 Perspectives

Since its invention ∼50 years ago, sequencing quality has improved while costs have decreased (Cheng et al., 2023; Satam et al., 2023). Sequencing will continue to evolve as existing technologies mature and new ones emerge, leaving technology selection a constantly evolving decision. Given the increased use of sequencing, public sequence data will likely continue to expand, a monumental resource to scientific discovery by secondary analyses. Now, after acquiring nucleic acid sequences, omics analysis can begin.

### 4.2 Sequence statistics

The first products of any omics analysis are generally sequence statistics, used for narrative or quantitative purposes (Figures 2B, 3A). For narrative purposes, read statistics are used to show that there is sufficient sampling to test a hypothesis (total bp sequenced per sample; Liu et al., 2015). For contigs, descriptive statistics are used to summarise assembly success: longest contig, total contig counts, N50, L50 metrics (Mikheenko et al., 2016). Genome statistics can indicate binning success: contamination in genomes (Bowers et al., 2017), genome size (Chklovski et al., 2023), and how representative the genomes are of a sampled community (percent of reads mapping to all genomes; Hauptfeld et al., 2024). These statistics should be generated any time that a sequence file is acquired or produced, with multiple tools available to streamline these calculations (reads: Nonpareil3, Rodriguez-R et al., 2018; contigs: QUAST; Mikheenko et al., 2016; genomes: BUSCO; Seppey et al., 2019; CheckM2; Chklovski et al., 2023). For quantitative purposes, sequence statistics are generally used to normalize count data against the length of the sequence and sequence library size, or to compare counts across or within datasets (simplified by efficient tools; SeqKit, Shen et al., 2024). For there to be any sequences to statistically summarise, we must first apply quality control standards.

### 4.3 Quality control

The quality of sequence files should be examined at the levels of reads (Figures 3B,K), contigs (Figures 3D,N) or genomes (Figure 3F) to increase confidence in any results.

#### 4.3.1 Contaminant removal

Non-target sequences (contaminants) should be removed from a sequence library before downstream analysis (Figures 3B,K,L). Contaminating sequences can originate from several sources, including defined organisms expected to be in the sample but not the target of inquiry (human DNA sequences in an analysis of the human skin microbiome) or incidental organisms that should not be in the sample (plasmid or bacterial DNA in a reagent solution). Defined contaminants can be removed by aligning the new reads against a contaminant’s genome or transcriptome and removing reads that align to the contaminant (Lataretu et al., 2025). Incidental contaminants are ideally detected by sequencing negative controls (where no sequences are expected) from various steps of sampling and sequencing preparation, with any recovered sequences representing potential contaminants (Fierer et al., 2025). These sequences can be classified as contaminants based on statistical probabilities (Davis et al., 2018) or taxonomy (based on taxa known to contaminate molecular biology reagents; De Goffau et al., 2018). In cases of limited contamination, it is recommended to remove (and report) potential contaminant sequences (Clum et al., 2021) while samples with rampant contamination may need to be discarded entirely (Fierer et al., 2025). These removed sequences may represent true biological signals, as knowledge of any biological system is often incomplete–with any decontamination balancing description of true novelty and cautious interpretation of data. Now we discuss standard quality control methods in read data.

#### 4.3.2 Reads

Evaluations of read quality should consider whether sequences are of: 1) high quality and 2) sufficient quantity for the planned analyses.

Assessments of read quality should consider both sequence length and confidence in base assignment. A sequence substantially shorter than expectations (relative to sequencing technology) may indicate a poorly sequenced molecule and should be removed (Martin, 2011). Base confidence (in FASTQ sequence files) is encoded by the quality-score (Q-score), estimating the probability that a single base in a sequence is correctly assigned (A,C,G, or T), with higher Q-scores indicating higher confidence (O’Rawe et al., 2015). Quality filtering reads first trims sequences to remove low quality (user specified Q-score) bases, with the whole sequence discarded if trimming shortens it past a minimum length (TrimGalore! – https://github.com/FelixKrueger/TrimGalore), a process that should be performed before assessing sequence quantity or performing other omics analyses.

The necessary number of reads is dependent on the type of sample, with more complex microbiomes requiring more sequencing than simple ones (Rodriguez-R and Konstantinidis, 2014). Sampling sufficiency can be assessed using rarefaction analysis, where cleaned reads are randomly subsampled, a metric of novelty calculated at each increment, and then plotted against one another (sequence diversity vs. read number; Rodriguez-R et al., 2018). If novelty saturates (asymptotes) with increased read number, most of the sequence diversity was captured, whereas a linear relationship–without saturating–indicates unsequenced diversity. Unrepresentative samples can be resolved with more sequencing, but if this is not possible, such samples can still provide useful–though caveated–information. Next, we discuss contigs.

#### 4.3.3 Contigs

Evaluations of contig quality should consider 1) assembly quality and 2) if the assembled contigs represent the sequenced reads.

Assessments of assembly quality typically consider the number of contigs, length of the longest contig and the metrics: L50 and N50. Acceptable values for the number of contigs and longest contig can vary depending on study goals and sequencing technology used, but large values for both metrics indicate a better assembly. The metric N50 calculates the length of the shortest contig at which all contigs as long or longer than the N50 value encompass 50% of the contigs–a weighted median contig length. Larger N50 values indicate that an assembly consists of longer contigs, generally indicating assembly success (International Human Genome Sequencing Consortium et al., 2001). The metric L50 represents the smallest number of contigs whose summed length constitutes half the total length of the assembly. A large N50 value combined with a smaller L50 indicates that the assembly is composed of a few long (likely data-rich) sequences (Bradnam, 2015). Though informative, using contig length to infer assembly quality requires caution, as these metrics are useful to compare assemblies against one another–especially when assembling a single genome–but in a mixed microbiome, small contigs are not necessarily a problem. Short (<2,000 bp) contigs can still provide valuable information and can be common for communities enriched in plasmids, viruses, mobile genetic elements, or low-abundance microbes (Maguire et al., 2020; Kieft and Anantharaman, 2022). These short contigs are often removed by default when binning genomes (Alneberg et al., 2014), with the justification that the average bacterial gene is ∼1,000 bp (Xu et al., 2006) and shorter contigs are unlikely to contain complete genes. This removal can discard valuable genetic context and should be performed with full knowledge of the risks for data loss.

Assessing the representativeness of assembled contigs for a microbial community often involves aligning the un-assembled reads to the assembled contigs (Aroney et al., 2025). The percentage of reads aligning to the contigs indicates how much of the original information is present in the derived contigs. If the percentage of reads aligning to contigs is high (>90%) then the contigs can be considered representative of the community while a low value (<50%) indicates that the contigs are not representative. In cases of unrepresentative contigs, the assemblies still contain useful information for individual genomes, but community-scale inference may require refining contig assembly (parameter optimization, more sequencing) or performing read-only analyses (see Section: 4.4 Read-based Marker Gene Analyses”). Next, we discuss quality control in genomes.

#### 4.3.4 Genomes

Genome quality is most often assessed by the metrics of “completeness” and “contamination”, for which there are published quality standards (Bowers et al., 2017). User-friendly tools exist to calculate both metrics, with new versions accounting for whole-genome features (Chklovski et al., 2023). For illustrative purposes, we will describe quality estimation using older methods–that use a constrained number of single copy marker genes–as they are more tractable for beginners (Parks et al., 2015). In the marker gene-based approach, genomes are screened for the presence of a set of single copy marker genes, with the assumption that a complete genome should have only one copy of each gene in the set. In this approach, completeness is estimated as the percentage of marker genes detected in the genome with contamination based on how many times a single copy gene was duplicated–potentially indicating errors in assembly or binning. Ideally, genome analysis would be performed on whole uncontaminated genomes, but this is often impossible due to limited sequencing data, requiring the use of incomplete or contaminated genomes. These imperfect genomes can still provide useful insights, if caveats in the interpretation of these data are acknowledged.

Despite the utility of standard quality metrics, there are cases where they are misleading. First, relying on simplifying metrics obscures reality. Computationally, a “complete” genome does not mean that the genome is “closed” or “finished” (i.e., represented by a single contig without gaps; Bowers et al., 2017) which is an even higher standard of quality. Further, many studies only analyze “high to medium quality” genomes (Bowers et al., 2017), potentially discarding other genomic data that does not conform to the expectations of “completeness”, including endosymbionts, plasmids, and viruses, all essential components of a system. Combining both challenges, obtaining a “complete” or “closed” genome cannot assess if a single organism contains multiple chromosomes (*Rhizobium*, Landeta et al., 2011) and/or nucleic acids from other sources (viruses, plasmids, endosymbionts). These shortcomings are systemic but can be overcome with intentional analysis. Obtaining closed genomes often requires focused efforts (long read and/or deep sequencing), while recovering overlooked plasmids and viruses can come from otherwise discarded data (Fogarty et al., 2024). Identifying which chromosomes, endosymbionts, plasmids, and viruses reside inside one organism requires sequencing single cells (i.e., single amplified genomes; Labonté et al., 2015) to gain a fuller understanding of their importance and functions. Now, we move on to discuss the use of marker gene surveys in omics.

### 4.4 Read-based marker gene analyses

Quality controlled reads can be used to provide insight into the taxonomy or functional potential of an organism or community through the analysis of marker genes. Genes are considered “markers” if they are involved in metabolisms of interest (e.g., *nifD*: encoding the nitrogen fixing Nitrogenase molybdenum-iron protein alpha chain or *mcrA*: encoding the methane producing Methyl-coenzyme M reductase I subunit alpha) or can be used to reconstruct evolutionary relationships (e.g., 16S rRNA gene or *rpoB* encoding the beta subunit of bacterial RNA polymerase). Such genes are typically well represented in existing databases, serving as useful references for comparison (phylogenetic analysis). Marker gene analyses most commonly use targeted amplification and sequencing (Figure 3C). As an example of taxonomically informative marker genes, Lozupone et al. (2013) used 16S rRNA gene amplicons sourced from global sequencing of human microbiomes to identify forces structuring communities including disease status and body site. Surveying function, Dumont et al. (2014) used gene amplification for *pmoA* (particulate methane monooxygenase, beta subunit) to identify the presence of methanotrophic bacteria and delineate phylogenetic clusters. Somewhat less commonly, marker genes can be recovered from untargeted metagenomic and transcriptomic sequencing (Figure 3K), where reads are aligned to marker gene databases, with confident read alignments to a gene indicating its presence and abundance. For example, Maritan et al. (2025) searched marine sediment metagenomes for metabolic marker genes involved in aerobic and anaerobic metabolisms in coral reef sediments. For taxonomy, Urayama et al. (2024) surveyed the prokaryotic taxonomic composition of metagenomes from several hot springs using fragmentary rRNA sequences before digging deeper into the sequences of co-existing viruses. Both amplification and genome/transcriptome applications are appropriate for targeted questions that involve the constrained goals of identifying specific metabolisms or taxa. While the genomic/transcriptomic approaches have the advantage of being able to initially query the whole dataset (Hauptfeld et al., 2024) for specific genes and later studying more detail though assembly and binning, which we will discuss next.

### 4.5 Contig assembly and analysis

Contig assembly aims to reconstruct longer and more information-rich sequences from shorter reads. This process entails two steps: 1) normalization and 2) assembly.

#### 4.5.1 Read normalization

Read normalization (Figure 3B) reduces the computational burden of contig assembly by limiting the amount of data passed to the algorithm. This is achieved by subsampling redundant sequences and removing low abundance sequences (that are unlikely to assemble). Normalization is appropriate for diverse (e.g., sediments; Maritan et al., 2025) and simpler (e.g., hot springs; Colman et al., 2024) samples. Read normalization is straightforward to implement with tools like bbnorm, developed by the Joint Genome Institute (“https://sourceforge.net/projects/bbmap/”), where read data is input and normalized, with the output generally ready for contig assembly.

#### 4.5.2 Contig assembly

Assemblers (Figures 3D,N) use short reads to reconstruct longer sequences (DNA or RNA). Detailing assembly algorithms is beyond the scope of this review (described in Ekim et al., 2021; Yang et al., 2021), but illustratively, assemblers look for overlap between reads and use this overlap to create longer and longer sequences (Ayling et al., 2020). There are two major classes of assembly: 1) guided and 2) *de novo*.

Guided (i.e., reference-based) assembly aligns reads to sequences from related organism(s), serving as a scaffold to guide placement of the read data. These guide sequences should be sourced from organisms closely related to those in the reads and can be a reference genome (i.e., reference guided assembly Lischer and Shimizu, 2017); or long read data from the same sample (i.e., hybrid assembly, Antipov et al., 2016).

*De novo* assembly has two varieties: 1) individual, and 2) co-assembly. In individual assembly, reads from a single sample are assembled into contigs. In that case, all assembled contigs are by-definition present in that sample. In co-assembly, reads from similar samples (soils from the same site; Riley et al., 2023) are combined and then assembled as a single dataset. This is often done with the aim of generating contigs from lower abundance organisms (Riley et al., 2023). Some resultant contigs from a co-assembly might not be present in all the source samples, but presence/absence can be determined by quantifying abundances of the contigs in the sample (see “Section 4.10 Count Data”). In co-assembly, reads should be normalized after combining samples, thereby potentially retaining low abundance sequences that might have been removed in individual normalization. To maximize contig recovery, it is possible to assemble contigs using both individual and co-assembly approaches and then later remove any duplicated sequences (see “Section 4.7 Sequence dereplication”).

These contigs can be used to study standalone genes, plasmids, or viruses. For example, contigs were used by Priest et al. (2025) to identify seasonal patterns of functional potential in the Arctic Ocean, while Fogarty et al. (2024) searched for novel plasmids in human gastrointestinal tracts, and Zhong et al. (2024) identified viruses encoding methane cycling genes. Though these contig-analyses are useful, the most common use of contigs is binning into genomes.

### 4.6 Genome binning and analysis

Creating genomes from contigs (Figure 3F) involves grouping contigs into distinct, taxonomically coherent “bins”. These bins represent draft genomes that must then be evaluated for quality, completeness, taxonomy, and function. Genomes binned using sequences from pure culture isolates or single amplified genomes (SAGs) may be considered “strains” (Conrad et al., 2022). In contrast, genomes binned using sequences from community sequencing are called metagenome-assembled genomes (MAGs) that often represent consensus sequences from multiple closely related strains sharing similar but non-identical genomes (Meziti et al., 2021).

#### 4.6.1 Binning contigs

Binning programs (binners) typically separate contigs into bins based on shared genomic features and read depth (Bowers et al., 2017). Binners assume that intrinsic genomic features, such as GC content and oligonucleotide (i.e., k-mer) frequency, are consistent across a genome (Bussi et al., 2021), allowing an initial univariate (GC) or multivariate (k-mer composition) separation of contigs into clusters. These initial clusters can be refined with the additional assumption that contig read depth–as measured by the number of reads aligning to each assembled contig–is also consistent for all contigs for a given genome (Sharon et al., 2013). Each individual binning tool generally implements all or some of these approaches (Alneberg et al., 2014), creating draft genomes that can be further refined, annotated, and compared.

#### 4.6.2 Genome improvement

Maximizing the accuracy and information content of individual genomes can be done by selecting the highest quality genomes generated using multiple binning programs (refinement) and reassembling high-quality genomes (reassembly).

Refinement starts by using multiple binners to generate somewhat redundant bins. The resulting genomes from each of these binners are compared to find the highest quality genomes with the highest completion and lowest contamination values. Each chosen genome is placed in a final bin set, often with a cleaning step to ensure each contig is only found in a single–highest quality–bin (Uritskiy et al., 2018). Reassembly uses high quality (quality controlled, refined, and/or dereplicated) genomes to try to re-generate these genomes with even better contigs. This involves aligning the original quality-controlled reads to each genome to “isolate” sequences for an organism of interest. These reads can then be re-assembled using a non-metagenome assembler (SPAdes instead of metaSPAdes; Uritskiy et al., 2018), repeating alignment and re-assembly until achieving a genome with the greatest completion and smallest contamination values possible (Kitzinger et al., 2020).

#### 4.6.3 Shortcomings and hazards

While useful and widespread in omics, genome binning does have shortcomings. First, not all sequences can be binned. Binning relies on high quality assemblies that can be grouped based on sequence similarity–which requires that disparate parts of a single genome have similar sequence characteristics (Nelson et al., 2020). This assumption may not be true for genome fragments that have been acquired by horizontal gene transfer (HGT), carrying sequence characteristics different from those of the recipient’s genome (Mise and Iwasaki, 2022). Similarly, genetic elements not incorporated into a genome (such as plasmids and viruses; Eren and Banfield, 2024) or second chromosomes (Landeta et al., 2011) do not meet the assumptions of binners. Without contiguity and/or sequence similarity to the focal chromosome, HGT-derived genes, mobile genetic elements, and second chromosomes may be erroneously separated from their true genomic neighbors (Maguire et al., 2020). Second, intergenic or non-protein coding genomic regions (ribosomal RNA operons) and genomic regions with repetitive sequence features, are often challenging to assemble or bin correctly and are underrepresented in genomes (Mise and Iwasaki, 2022; Wilbanks et al., 2022). Third, in samples containing multiple closely related organisms, genome-approaches collapse strain-level microbial diversity, blurring intra-species genomic boundaries (Wilbanks et al., 2022) and obscuring genomic novelty. In these instances when binning excludes sequences or blurs organism boundaries, analysis of binned data may lead to inaccurate measurements of community-level diversity, fail to detect certain taxa or functions, and provide an incomplete view of the genomic environment of cells.

Many of these shortcomings can be minimized. Complex and HGT-derived genomic sequences can be definitively linked to their genomes using long-read sequencing to sequence across ambiguous genome space (Wilbanks et al., 2022). Capturing the diverse genomic material (chromosomes, plasmids, viruses) in a single cell can be achieved with single amplified genome (SAG) sequencing. SAGs also provide strain-level genomes, helping to resolve heterogeneity among closely related genomes. Once reads, contigs, and genomes are generated, they can be simplified by dereplication before analysis.

### 4.7 Sequence dereplication

Sequence redundancy is common from reads to genomes and can be removed to reduce computing requirements or analytical repetition. Dereplication calculates sequence similarity between sequences (reads, contigs, and genomes) with an array of programs (VSEARCH, Olm et al., 2017; Rognes et al., 2016; MMSeqs2; Steinegger and Söding, 2017; CD-HIT; Fu et al., 2012) and then uses similarity cutoffs to create clusters of similar sequences (sequence clustering; Box 1). Once sequence clusters are identified, the highest quality sequence in each cluster can be extracted and used as a representative for all other sequences in its cluster. In read data, clustering is most frequently seen in taxonomic marker gene analysis using operational taxonomic units (OTUs; Hughes et al., 2001; Box 1). Contig clustering often takes the form of gene catalogues, where protein coding sequences on a contig are clustered, often principally by taxonomy and then by sequence similarity (Muratore et al., 2022; Priest et al., 2025). Finally, genome clustering is most often used for dereplication of entire genomes (Figure 3F). In all these use cases, dereplication by sequence similarity is a powerful and unbiased approach to simplify similar sequences. These sequences are now ready for taxonomic classification and functional annotation.

### 4.8 Taxonomic classification

#### 4.8.1 Roadmap for implementation

Many analyses aim to connect sequences with taxonomic labels (reads, Figures 3C,K; contigs; Figures 3E,N; and genomes; Figure 3G). Taxonomic classification often relies on aligning an unknown query sequence against a database (untargeted: NCBI nt/nr; or molecule-specific: SILVA rRNA) of sequences with defined taxonomies (i.e., subject sequences), with the query inheriting the taxonomy of its–sufficiently similar–best aligned subject sequence. A common implementation of taxonomy-by-alignment involves using the NCBI BLAST webserver (Camacho et al., 2009; https://blast.ncbi.nlm.nih.gov/Blast.cgi) to align a query against one of multiple databases, providing accessible fast taxonomies. Alignment-based classification is effective (Jain et al., 2018) but can be supported by estimating evolutionary divergence of the query sequence compared to taxonomically resolved homologues. These homologues are selected to include both close and distant relatives of the query and used to construct a phylogenetic tree (see “Section 4.11 Phylogeny”). In this method, the query inherits the taxonomy of its–sufficiently similar–closest neighbor. Implementing phylogenies is straightforward with multiple tools for automated (GTDB-tk, Chaumeil et al., 2022) and semi-automated (PhyloPhlAn, Asnicar et al., 2020; MarkerFinder; Martinez-Gutierrez and Aylward, 2021) phylogenetic classification, providing broad access.

Though both direct-alignment and phylogenetic placement are applicable to read, contig, and genome based-analyses, longer sequences encode more evolutionarily relevant information and thus provide better taxonomic resolution than shorter ones. This is of limited concern for genome-based analyses (containing Megabases to Gigabases; Milo and Phillips, 2015) but can produce less reliable taxonomy for reads (100–250 bp; Hauptfeld et al., 2024). Read length limitation can be overcome by using reads to reconstruct and classify the more informative contigs and genomes, then assigning the constituent reads the taxonomies of their contigs and genomes. Ultimately, this multi-step classification combines the taxonomic clarity of genomes and the community representation of reads (see “Section 4.4 Read-based Marker Gene Analyses”) to achieve a high-quality understanding of the sequenced community (and is implemented in open access tools; Hauptfeld et al., 2024).

#### 4.8.2 Shortcomings and hazards

A note for users, the quality of taxonomic classification is dependent on the completeness of the reference database. Under ideal circumstances, database subject sequences originate from an isolated, living specimen providing a confident association between database taxonomy and a living organism. As sequencing captures more diversity than exists in-culture, connecting a query sequence to a type specimen is often not possible, instead requiring comparison to uncultured sequences (MAGs; Murray et al., 2020). This means that assigning taxonomy to divergent organisms requires more effort (phylogenies; Eme et al., 2023) than in organisms closely related to models (*E. coli* and *Staphylococcus aureus*), potentially requiring the creation of new taxonomic groups (Rinke et al., 2013; Murray et al., 2020). Another potential concern for assigning taxonomy is the influence of horizontal gene transfer. The exchange of genes between organisms (bacteria-bacteria, Tschitschko et al., 2024; bacteria-virus; Li et al., 2025; bacteria-eukaryote; Porras et al., 2024) can obscure the evolutionary lineage of any one sequence. Disentangling the current genomic placement–and taxonomy–of any gene generally requires situating it in a complete, contiguous genome.

Taxonomy is a useful, but incomplete classification of living organisms (Aldrich, 1927; Staley, 2009; O’Brien and Luo, 2022). Indeed, ecosystem-scale analyses (biogeochemistry) sometimes pay little to no attention to taxonomy, focusing only on functions encoded in nucleic acids. In aid of both taxonomy-agnostic or -informed analyses of encoded functions, we will next discuss functional annotation.

### 4.9 Function annotation

The encoded biochemical outputs (expressed RNA and translated proteins) are the focus of many analyses. The act of assigning inferred function to a sequence is called annotation. Functional annotation of reads (Figures 3C,K), contigs (Figures 3E,N), or genomes (Figure 3G) predicts the potential cellular activities of nucleic acid molecules (rRNA, tRNA) or–most commonly–of encoded proteins. Like taxonomic classification, functional annotation compares a query sequence against a database of annotated reference sequences. Under ideal circumstances, prior experimental studies have confirmed the biochemical function of molecules encoded by the reference sequences.

Annotations of non-protein coding regions are identified directly from nucleic acid sequences (rRNA: Barrnap, https://github.com/tseemann/barrnap; tRNA: tRNAscan, Lowe and Eddy, 1997) while protein coding genes are either identified directly from reads or from identified protein coding regions (from reads, contigs, genomes). Identifying protein coding regions (i.e., open reading frames, ORFs; Box 1) searches for their molecular characteristics (e.g., start and stop codons; tools: Prodigal, Hyatt et al., 2010; FragGeneScan; Rho et al., 2010) outputting likely protein-coding sequences for use in homology searches.

Functional annotation is performed as either a targeted or untargeted search. A targeted search focuses on dozens of genes of biogeochemical or ecological significance (Leung and Greening, 2020; Zhou et al., 2022), identifying the potential for a microbiome to perform specific functions of interest. Targeted searches can input short reads (Dragone et al., 2022; Bertagnolli et al., 2023) or open reading frames (Priest et al., 2025). This approach can be used to quantify the presence of catalytic genes in a sample. For example, Dragone et al. (2022) searched Antarctic soil metagenomes for genes involved in trace gas cycling to quantify the genomic potential of the entire microbiome to utilize trace gasses across multiple environments. Targeted searches are also useful as an initial screening of large genomic datasets (reads to genomes) before digging deeper. For example, Speth and Orphan (2018) were interested in the diversity of methanogens across thousands of publicly available metagenome datasets. To save computing time, they pre-screened datasets for the presence of diagnostic methanogen gene *mcrA* (Methyl coenzyme M reductase), only assembling contigs and binning genomes from *mcrA* positive datasets.

Untargeted searches do not have specific genes of interest, instead aiming to annotate as many sequences as possible. This approach is best suited to ORFs because they contain enough genomic content to be confidently annotated against hundreds of thousands of reference genes. This endeavor often starts off semi-targeted, using tools searching for tens of thousands of specific genes (Prokka, Seemann, 2014; KofamScan; Aramaki et al., 2020). The sequences that remain un-annotated after this first pass may still be amenable to annotation and can then be queried against even more comprehensive databases (NCBI nt/nr, UniProtKB). If homologs to these sequences cannot be identified, a cautious approach is to designate such ORFs as “proteins of unknown function”, or “hypothetical proteins”. The functions of these hypothetical proteins may be inferred based on the functions of nearby sequences (within the same operon; Mihelčić et al., 2019) or demonstrated using non-omics approaches (biochemistry and cell biology; discussed below). An untargeted approach will generate a lot of annotations and is most tractable when constraints are applied to its analysis. One way of constraining the analysis is by examining only a few genomes in-depth. For example, Mitchell et al. (2024) sought to examine gene expression for a single bacterium, using five semi-targeted tools and the NCBI non-redundant protein database to annotate the genome. Another method to constrain the large volume of information from an untargeted analysis is to use an annotation system with a simplifying gene hierarchy (ontology; Box 1). For example, Kelly et al. (2019) annotated seawater metagenomes with the SEED subsystem database–grouping genes by functional categories–which they used to collapse annotations into functional groups, making the analysis of thousands of sequences tractable.

Mechanistically, functional annotation often relies on sequence alignment or Hidden Markov Model (HMM) searches. Alignment compares a query sequence (nucleotide or translated amino acid) to a functionally annotated subject sequence, identifying regions of sequence similarity. If the two sequences are sufficiently similar, the query sequence is assigned the annotation of the subject. The most common sequence aligners are those of NCBI’s Basic Local Alignment Search Tool, which work by finding identical sequence fragments (substrings) between a query and reference and then expanding the alignment outward from the region of identity (BLAST, Camacho et al., 2009). BLAST searches can be performed via a web interface that accesses NCBI’s servers directly or–more efficiently–using BLAST software installed locally. BLAST-based homolog identification can be computationally intensive but is generally accurate (Al-Fatlawi et al., 2023). In the decades since BLAST was introduced, other alignment alternatives have been developed, with many of these being faster and equally or more accurate (DIAMOND, Buchfink et al., 2015). HMM-based approaches use databases of homologs to build a profile/model for a protein or protein domain of interest. This HMM profile contains features (the probabilities of different amino acids at different positions) intrinsic to the protein or protein family and can be used to search a sequence dataset to identify putative homologs with high confidence (details of HMMs reviewed in Mor et al., 2021). HMMs can be more sensitive than BLAST searches in identifying distant homologues (Kirsip and Abroi, 2019) but require training on high quality sequence data. Fortunately, several repositories of pre-trained HMMs are available (TIGRFAM, Haft, 2001) with some integrated directly into annotation tools (KofamKOALA, Aramaki et al., 2020). Though not widespread yet, attention-based artificial intelligence also holds great potential for functional annotation (Hwang et al., 2024) and prediction (Jumper et al., 2021) but is beyond the scope of this brief overview.

An important note, confidence in any gene’s annotation is a balance between effort and confidence. Many genes can be annotated quickly–to a high degree of confidence–but approaching “proving” that an encoded gene can perform a function requires increasing effort. This may require narrowing the focus from many (10,000+) to a few (1–10) genes, eventually departing from omics altogether for the domains of biochemistry and molecular genetics (Table 2). Protein purification or heterologous expression should only be used for absolute proof, as–in most cases–automated gene annotation or simple phylogenies are sufficient to hypothesize the functions of a gene. Solid annotations lay the foundation to compare gene prevalence, abundance, or expression between systems via quantification.

**TABLE 2:** **Increasing confidence in functional annotation is increasingly time intensive and eventually requires non-computational approaches**: A simple workflow for increasingly confident annotations with steps, actions, realistic number of sequences to analyze, interpretations, and examples in the literature.

Steps	Action	Number of sequences analyzed with this technique in one study	If confirmatory, what does this tell you?	Published example
1: Identify likely homologues	Identify candidate homologues (BLASTn/p, HMM)	10,000+	Target sequence is sufficiently similar to known sequence to be a homologue, though may include false positives	Screen thousands of genes (Anantharaman et al., 2016)
2: Contextualize phylogenetically	Phylogenetically place gene of interest against high confidence (SwissProt) gene sequences	10s	Target sequence is situated with other sequences known to perform the function of interest	Tree genes of interest (Graf et al., 2021)
4: Identify essential motifs and structures	Identify key motifs (Pfam) and structures (AlphaFold)	10s	Target sequence possesses necessary architecture for claimed function	Identify functional residues (Porras et al., 2024)
5: Assay with biochemistry and molecular biology	Knockout or clone gene of interest and biochemical assay	1–2	Target sequence performs the assayed function	Clone gene (Tsementzi et al., 2016)

### 4.10 Count data

Quantifying omic features in a dataset (reads, Figures 3C,K; contigs; Figures 3E,N; or genomes; Figure 3G) uses read data. Read quantification involves counting reads of a given type (reads aligning to marker gene regions), typically followed by normalization to sequencing effort (e.g., Reads per Megabase of sequencing). Contig or genome quantification requires aligning reads to these longer sequences, typically followed by normalizing for contig/genome length and dataset size (e.g., Reads Per Kilobase of Contig per Megabase of sequencing). It should be noted that alignment-based quantification may overestimate sequence abundances with methods developed to counteract this (TAD80; Viver et al., 2021).

Count data are prevalent across genomic studies, estimating the abundance of genes (Dragone et al., 2022; Maritan et al., 2025; Ricci et al., 2025) and microbes (Steinsdóttir et al., 2022; Shoemaker et al., 2024) in a sample. In transcriptomics, cDNA-derived reads are aligned to a reference sequence (genome, Bertrand et al., 2015; or assembled transcript; Sorek et al., 2018) to estimate transcription levels of genes. Metagenome and metatranscriptome studies can also quantify exact numbers of transcript molecules per amount of sample or per gene copy number. This is most precise when mRNA or genomic DNA standards are spiked into samples (Moran et al., 2013; Nowinski et al., 2023) but can also be estimated by normalizing gene expression to measured biochemical properties (expressed mRNA per gram soil; Söllinger et al., 2018; Täumer et al., 2022). This allows precise quantification of omics data and can be especially useful for estimating changes in metabolic activity.

These count data of gene abundances and expression levels provide a basis for hypothesizing about the function of a system but generally require other methodologies for confirmation (quantitative PCR, cell counts, chemical measures, or cell culture).

### 4.11 Phylogeny

Because phylogenetic inference is essential to taxonomic and functional omics analyses, we will briefly summarise the methods for phylogeny construction here. However, we note that this is only a primer and does not cover all the details needed to correctly perform these analyses. For more in-depth discussion, we direct readers to excellent reviews describing the principles and tools for phylo-genetics/-genomics (Kapli et al., 2020; Steenwyk et al., 2023).

A phylogeny or phylogenetic tree (Figure 3J) shows the evolutionary relationship of a focal sequence relative to reference sequences. Interpreting a phylogeny involves examining two key features: 1) topology and 2) branch length. Topology describes the shape of a phylogenetic tree, including branching patterns and clusters of sequences (Kapli et al., 2020). Assuming there is statistical support (via bootstrapping) for the groupings in the tree, sequences clustering together is often used to support claims that a focal sequence shares evolutionary history with a taxonomic (Eme et al., 2023) or functional (Porras et al., 2024) group, permitting classification or annotation. Conversely, divergence between sequences can be used to delineate new taxonomic groups at coarse (Woese and Fox, 1977; Lane et al., 1985) or fine phylogenetic scales (Tsementzi et al., 2016) and follow up with the question: “what changes have accumulated between two diverging sequences” (e.g., individual sequences, Major et al., 2017; whole genomes, Conrad et al., 2022). Branch length–in a rooted tree–describes the distance from a phylogeny’s root to any tip, serving as a proxy for a lineage’s age. Time calibrated branch lengths (using dated fossils or geochemical evidence to contextualize divergence) provides insights into the exact timing of diversification (Damsté et al., 2004; LaJeunesse et al., 2018). Beyond describing divergence timing, branch lengths can be used to quantitatively assess how evolution drives ecological associations (Colman et al., 2024).

The creation of a phylogeny comprises three main steps: 1) sequence acquisition, 2) multiple sequence alignment, 3) phylogenetic inference (reviewed in Kapli et al., 2020). Both nucleic acid and amino acid sequences can be used for phylogenetic inference. It is common practice to use nucleic acids to resolve closely related organisms (due to more combinations available for nucleic acids to specify any codon than for amino acids) and amino acid sequences for more distantly related sequences, though nucleic acids and amino acids may provide similar resolution for distant relationships (Kapli et al., 2023).

The first step of sequence acquisition involves identifying sequences for phylogenetic reconstruction. This can be done manually (BLAST genomic sequences against a gene of interest reference database) or automatically (MarkerFinder, Martinez-Gutierrez and Aylward, 2021). If the analytical goal requires comparing homologs only, it may be necessary to remove potentially non-homologous–but similar–sequences identified by sequence searches. This step often requires manual inspection and can be time-intensive (reviewed in Kapli et al., 2020). The product of sequence acquisition–a set of confident homologues–is the starting point for the next step, multiple sequence alignment (MSA). MSA compares sequences to correctly orient homologous base or amino acid positions along the sequence. This results in a matrix in which the rows indicate sequences and columns indicate homologous positions in a sequence, with residues (bases/amino acids) shared among sequences at the same position often indicating shared ancestral patterns of sequence change. If creating a phylogeny from multiple genes, genes may be first combined (concatenated) and then aligned or first aligned separately and then concatenated, in both cases creating an MSA supermatrix. The accuracy of phylogenetic reconstruction depends intrinsically on the accuracy of the MSA. Therefore, any MSA should be manually examined after the (typically) automatic step of alignment, potentially to verify strandedness of homologues (so as not to mistakenly compare palindromic regions), remove sequences that align poorly or with high percentages of gaps, or mask ambiguously aligned regions (Kapli et al., 2020). Finally, phylogenetic inference involves generating a bifurcating tree that estimates evolutionary relationships based on shared residues in the MSA and a model (set of assumptions) about the process of sequence change. This step can be performed by creating and merging multiple trees from each of the aligned genes or a single tree from the gene supermatrix. The methods for constructing trees are diverse and vary in the extent to which they estimate and incorporate parameters describing the evolutionary process and, consequently, the time and computational resources required for the analysis (Kapli et al., 2020).

Phylogeny, and all the previously described tools, were given only a brief treatment. Our aim was to provide a foundation for readers to seek out more in-depth guides as needed. We will end our discussion of tools by highlighting new frontiers for omics application.

### 4.12 Contextualizing across datasets, time, space, and conditions

Individual tools are essential to produce the core omics data products, but once these data are produced, an omics scientist has the freedom to use these results to answer any number of scientific questions. We suggest that omics users make full use of publicly available databases to place their results into larger contexts (Figures 3H–J). Using public data, a researcher can compare their sequences against other similar (or different) studies to identify: shared or disparate trends (meta-analysis: Thompson et al., 2017; Kumagai et al., 2018; Ruff et al., 2024), reconstruct evolutionary histories (phylogeny: Hug et al., 2016; Eme et al., 2023), spatial distributions (biogeography: Härer and Rennison, 2023; Zhou et al., 2024), or driving environmental factors (modeling: Louca et al., 2016; Lui et al., 2021; Ramoneda et al., 2024; Chuckran et al., 2025). Each of these contexts is a discrete field with its own norms and tools beyond the scope of this review. In any case, situating omics findings in a broader context is almost always a worthwhile exercise that generally increases the utility and impact of omics research. We conclude this review with some final suggestions for new practitioners from our own experiences learning and teaching omics.

## 5 Tips for new practitioners

Starting to perform bioinformatics is formidable with layers of challenges. First, there are the concrete ones, learning how to code and manage terabyte sized sequencing datasets. Second, there is the conceptual task of designing workflows to generate useful results. Once these challenges are cleared, there remains the most formidable challenge, performing scientifically meaningful “experiments” on the computer. Thus far, this review has focused on the conceptual task of designing workflows for useful results. We will end with some suggestions for future work and literature to handle the fine details of coding and the broader issue of asking meaningful scientific questions.

### 5.1 Bioinformatics advice

#### 5.1.1 Opportunities for further training

Readers of this review should leave with an understanding of the motivations and methods of nucleic acid omics. For some readers, this review will be sufficient for their goals of digesting the “methods” sections of manuscripts, while others seeking to analyze data independently will need more specific training.

For those looking for additional training, we recommend three types of resources ordered from most accessible to most specialized. First, for guided exposure to using real data to run specific omics analyses, we recommend computing workshops or courses (in-person or online). Such workshops can be broad (binning MAGs) or specific (machine learning for protein prediction), providing an opportunity to develop a range of skills. Second, for self-guided learning of specific computing topics (read mapping to quantify transcripts, identifying viruses in omics data), we recommend online tutorials. These tutorials can be standalone websites (often the online material from a prior workshop) or published as part of a manuscript (e.g., Coenen et al., 2020). Tutorials are incredibly useful for users that can read and write some code (see “Section 5.1.2 Scripting”) and want to see how specific analyses are run, often demonstrated by analyzing subsampled real datasets. Finally, when trying to implement a specific tool (often found through a workshop or tutorial), we recommend reading the tool’s official documentation. This documentation often exists in two forms. First, many tools are announced with a publication describing their construction and general uses–which is useful for an overview but may overwhelm early omics users with technical information. Second, each tool generally includes a manual written for practical implementation. This may be as simple as a “README” text file included in the downloaded source code or as involved as a dedicated website to explain the uses and functions of the tool.

Building on the foundation of knowledge from this review, early-stage omics users will be able to acquire and integrate additional training to analyze data independently.

#### 5.1.2 Scripting

To interact with omics datasets, users can begin by using software that does not require much coding experience (Genious, Galaxy, Kbase), though accessing the full capacity of omics datasets requires learning to code (but does not require the skills of a professional programmer).

Scripting is the computing equivalent of pipetting in a lab, and aspiring bioinformaticians should be able to write and read code in Bash and R or Python. Bash is the basic language for performing omics analyses, used to interface with high performance computing clusters and run bioinformatics programs. Though Python can be used to write standalone programs, we suggest learning Python or R for their capacities to manage spreadsheets, perform statistics, and make plots. These tools take longer than Excel to master, but they quickly outperform it in flexibility, speed, and reproducibility. We suggest that bioinformaticians learn how to use either R or Python, as it is unlikely that knowing both will be essential, and if more languages are needed, they can be learned (Schloss, 2020). To learn coding, there are lots of online resources, with more available every day. For bash, we recommend chapters from the book *Practical Computing for Biologists* (Haddock and Dunn, 2011) pertaining to bash for a basic overview of some core commands and syntax and immediately applying it on real data to get a feel for its use. For R, the free online textbook *R for Data Science* (Wickham, 2023: https://r4ds.hadley.nz/) is an approachable read, organized to be practical and user friendly. For Python, we recommend the interactive free courses offered on Codeacademy (https://www.codecademy.com/). Though bash, R, and Python have been mainstream tools for decades (and may remain so), the scripting toolkits available and the resources to learn them will change over time, which should inform the training tools selected.

If coding is like pipetting, writing code with AI is like operating an automated liquid handler. Automating lab work may aid a novice wet lab scientist, but scientists with hands-on experience will better understand how to creatively and effectively implement such automation. AI support in bioinformatics works better with the specific vocabulary and perspectives that come from already knowing how to write code and manipulate data. That being said, we wholeheartedly recommend AI coding tools to help write tedious scripts (loops), make existing scripts more efficient, installing tools, debugging and explaining code. In any case AI users should be updated on best practice recommendations and publishing requirements (Buriak et al., 2023; Blau et al., 2024), they will change over time. In any case, a bioinformatician who knows what they want out of a workflow will be better able to get it with whatever basic or advanced tools they bring to bear.

#### 5.1.3 Local data organization

Data organization should be a primary directive. Files will always need naming and directories (folders) will always need organizing. We suggest you adopt a simple filing system (Noble, 2009) and adapt as you see fit. Do not proceed without some kind of system, as impromptu “organization” will eventually accumulate into an unwieldy mess. Two places where poorly organized files can cause major trouble are raw sequencing files and scripts.

All new sequence data should be placed in a clearly marked and backed up location (if working on a team, this original data should be in a shared “group” directory, not on the bioinformatician’s personal drive). This directory should have an informative name that contains the elements required to understand what is inside, for example,: sequencing run ID, sampling date, sequence type (genome, transcriptome, amplicon …), geographic source (country, ocean basin, cell culture collection …), and sequencing target (host organism, enrichment culture, soil …). In this directory, it is useful to have two sub-directories for reads. First the minimally processed reads from the sequencer (e.g., “o01_raw_reads”) and a second quality-controlled set (per “Section 4.3 Quality Control”; e.g., “o02_trimmed_reads”) as these processed reads will be used by multiple steps in any analysis and should be easily accessible. A file name tip: use character delimiters (e.g., “ . ”, “_”) to separate phrases in a file name instead of spaces (“ ”), to avoid problems later while scripting.

Next, the scripts that are written to analyze an omics dataset should be organized to allow the bioinformatician (or anyone else) to follow the workflow and backed up to prevent loss. Writing individual scripts for each step of an analysis is a good habit to keep the workflow clear and easily debugged. We recommend naming files sequentially so that auto-sorting arranges them logically (e.g., “o01_read_trimming.sh”, “o02_read_normalization”, “o03_assembly”).

Again, the raw sequence data and scripts should be backed up to prevent loss, as they are the minimum information required to regenerate all results.

#### 5.1.4 Public databases

One beautiful aspect of bioinformatics is the interoperability of sequences from diverse sources. The base FASTA format–adopted in 1985 and used today–has ensured that almost all sequence data uses consistent formatting (Wright et al., 2024). This consistency allows straightforward comparison of new data to archived sequences in repositories. Learning the major databases often involves talking to other scientists and looking at the methods of published papers, but once identified, these resources can easily provide sequences to contextualize new data or to supply material for meta-analysis (general: NCBI SRA, Leinonen et al., 2011; task specific: Tara Oceans, Sunagawa et al., 2020). Some tools exist to make these database searches easier (taxonomy browser, Parks et al., 2018; metagenomes pre-screened for community composition; Woodcroft et al., 2025; NCBI webtools; Sayers et al., 2024) though efficient use of these resources generally requires familiarity and practice. This practice is beneficial for any omics scientist, for a bioinformatician with databases is never without samples.

#### 5.1.5 Responsible data sharing and reproducible analyses

Thus far we have discussed scripting, data organization, and public databases from the perspective of how they benefit the reader of this review. Now, we will discuss the responsibilities of omics users to the broader scientific community–focusing on practices that ensure that published data and results are useful as long as possible. At its core, this means ensuring that raw data from published research is usable in the future and the results from a manuscript can be reproduced.

In an omics context, the space available in a manuscript is often too small to provide all the relevant data (sequences or environmental measures) for long-term use, and the researcher must then rely on external resources to ensure the information is accessible. To provide a brief coverage of the issue, we will discuss data archiving and reproducible analyses (though this coverage is necessarily incomplete and the reader should spend more time on these important topics).

##### 5.1.5.1 “FAIR” data archiving

Though sharing published data is a long-standing scientific practice and many journals require that the raw (unprocessed) data supporting the paper is made available, there is a lot of variation in how “available” could be interpreted. To clarify this, Wilkinson et al. (2016) introduced an influential set of guidelines for data management and stewardship summarised in the acronym “FAIR”: Findable, Accessible, Interoperable, and Reusable. We will describe some practices for “FAIR” data management in omics, though we will not cover each element of the acronym specifically and readers should spend more time leaning about the specific guidelines, especially before archiving their data (reviewed in Carballo-García and Boté-Vericad, 2022).

The foundation of omics data archiving requires that primary data files (unprocessed reads, assembled sequences, environmental measures) are available to other scientists, which is generally achieved by depositing data in public repositories. To maximize the lifespan of deposited data the repository needs to persist over time. An excellent example of a durable repository is also the most used for accademic omics data. The International Nucleotide Sequence Database Collaboration (INSDC) is an international effort to capture, preserve, and present nucleic acid sequence data for the “permanent scientific record” (Karsch-Mizrachi et al., 2025). This collaboration has operated for over 40 years, supported by the United States of America (National Center for Biotechnology Information; NCBI), Europe (European Molecular Biology Laboratory-European Bioinformatics Institute; EMBL-EBI), and Japan (DNA Data Bank of Japan; DDBJ). In this collaboration, data deposited to any participant (NCBI’s Sequence Read Archive, EMBL-EBI’s European Nucleotide Archive, and the DDBJ’s Sequence Read Archive) is exchanged with the others daily, ensuring global access and redundancy (Karsch-Mizrachi et al., 2025). Though other repositories exist for sequence data, the global support and historic record of the INSDC’s repository makes it one of the best options for most sequence data. Now we will turn our attention to the other essential elements of data archival.

One of the most critical elements of data archiving is that all primary data files must have unique and fixed identifiers (names). The exact names do not matter *per se* (though it is useful when these names have some intrinsic meaning; see “Section 5.1.3 Local Data Organization”) because all file identifiers should be explained by accompanying metadata. Metadata is essential to explain what is encoded in the primary data, thereby linking the primary data’s identifier to relevant descriptions of “what it is”. The types of collection descriptors are summarized in the widely used acronym “ISA”: Investigation (e.g., principal investigator, institutions), Study (e.g., location, organism, physical conditions, experimental condition), and Assay (e.g., type of nucleic acid sequenced, sequencing technology; Johnson et al., 2021). Lists of the minimum ISA descriptors needed for different types of primary data exist as “minimum information checklists” (examples at: https://fairsharing.org) and are often organized as standard templates (examples at: https://fairsharing.org). When filling out the relevant metadata, it is good practice to describe the data using well-defined hierarchical ontologies (e.g., taxonomic rank; examples at: https://www.ebi.ac.uk/ols4/) to avoid ambiguity, though new labels can be added as needed.

Finally, to be consistent with FAIR principles, both primary data and metadata should be encoded in widely available and commonly used file formats that describe the permissions or restrictions for reuse of the primary data, ensuring that anyone who retrieves the data will be able to read it and know how to use it appropriately.

##### 5.1.5.2 Reproducible analyses

Aside from sharing published data, omics users are expected to follow practices to ensure reproducibility of their results, allowing other scientists to double check their findings.

The most important elements of reproducibility are that other scientists have access to the raw data and the analytical tools used in the analysis. For access to raw sequence data most papers require that research generating new sequence data deposit it publicly (see “Section 5.1.5.1 ‘FAIR’ Data Archiving”) while those re-analyzing data list their sources (see “Section 4.1.3 Data Mining”). However, ensuring access to analytical tools is less regulated.

Though it is still common for scientists to use paid tools (“closed-source”; e.g., ArcGIS, MATLAB) for analyses, there has been a concerted effort by the broader scientific community to promote the use of open-source (free) software for research (Schloss, 2020), allowing broader access. For both closed- and open-source tools, it is expected that published manuscripts describe the software names and version used for each step in their analysis.

Finally, to ensure maximum reproducibility, omics users often publish the exact code used to run their analyses. This code is often written and organized with Git (a software for organizing code and handling version control) and made public through GitHub (a cloud-based service built on Git to share code). Publishing the code used for analyses is less critical when the analysis uses the default settings on published tools (which can be easily reported in a paper’s “methods” section) but becomes increasingly important when the research builds new tools and performs more complex analyses.

#### 5.1.6 Quantitative results: statistics, modeling, and guesswork

Omics analyses tend to present qualitative or descriptive quantitative results, rather than explanatory or predictive ones. Though the support of statistics or structure of modeling are not necessary for many good omics papers, the field of microbiology is ready to integrate these approaches more fully. There are examples where omics data is used to model biogeochemical processes (Louca et al., 2016; Täumer et al., 2022) and frameworks outlining how sequencing may be used in predictive models (Lui et al., 2021). More informally, scientists should practice making informed guesses, both qualitatively (from a literature base) and quantitatively (using benchmarked biological values; Fox, 2011; Milo and Phillips, 2015). Predictions (especially quantitative ones from rigorous modeling or informal estimations) are an excellent base from which to write clear, falsifiable hypotheses. Such hypotheses–especially when grounded by scientific literature–are foundational to any science (see “Section 5.2.1 Non-omics Literature and Toolkits”).

#### 5.1.7 Independent work

A final bit of omics advice is to become independent at performing the entire sample-to-analysis workflow: sample collection and preservation, nucleic acid extraction, sequencing prep (though we suggest out-sourcing sequencing to full-time professionals), to most omics analyses. This capacity simplifies troubleshooting and affords more control over the generation of any omics data. This capacity makes a scientist more independent and useful, ultimately a better hire.

### 5.2 Non-computational advice

Any omics user exists in a larger scientific environment of non-users. To integrate smoothly into this wider non-omics world, we have included some non-computational tips.

#### 5.2.1 Non-omics literature and toolkits

Though this review is focused on the technical details of computing, omics (like other tools: purifying proteins, culturing cells, or collecting samples) is a means to an end. The end goal of scientific inquiry is to incrementally improve human understanding of how the world works. Studying biology requires specific biological (not just computational) knowledge to guide analysis. This is especially true for omics, as any sequencing produces potentially overwhelming quantities of data, and biological context creates order and provides needed direction.

Analytical direction generally comes in the form of a biological question: “what are the tradeoffs involved in capturing light energy?” (Kumagai et al., 2018), “what pH did life originate in?” (Colman et al., 2024); “how does habitat specificity affect global patterns of speciation?” (Sriswasdi et al., 2017); “how do viruses shape mammalian biology?” (Henriques et al., 2024). In the best-case scenario, a biological question is used to inform data collection and analysis. However, as biological systems are often incompletely characterized, omics must often be exploratory, sequencing poorly understood microbiomes. In these cases, a focused scientific narrative requires crafting a biological question retrospectively.

To this aim, an omics scientist should be comfortable with non-computational biological literature (ecology, redox, stoichiometry, developmental biology, physiology, oceanography, pathogenicity, biochemistry). The goal of any omics scientist talking to an expert in their biological field, should be to be seen as “one of us”. Further, it is important to become acquainted with non-omics tools, especially how they fill the gaps left by omics (qPCR, microscopy, rate measurements, stable isotope probing, enrichment cultures, isolation, knockouts, microcosms, transformation, protein purification) and as appropriate, add these tools to one’s repertoire. A scientist that understands how omics fits into a constellation of other tools will be better equipped to plan research and identify tractable next steps (even if they never intend to do it themselves, it will help them find collaborators, see “Section 5.2.2 Networking”).

#### 5.2.2 Networking

Well-read and self-aware omics scientists should see themselves as a part of a global community with shared questions and aims. Tapping into this community to access the knowledge and skills of other scientists requires networking (i.e., making friends) in one’s focal-field and beyond. A network of familiar scientists makes scientific study more efficient, accessible, and enjoyable. Networking can be done anywhere scientists congregate (conferences, workshops, fieldwork, online) and is often more interesting and fruitful when it bridges diverse disciplines (biogeochemistry, ecology, biogeography, organismal biology) and departments (microbiology, ecology, earth sciences, geography, biochemistry, engineering). These connections can be used to identify good colleagues and great collaborators. Collaborating (working on the same projects together) with non-omics scientists will be far easier if the omics-scientist understands diverse methodologies and can effectively communicate what omics can and cannot do (see “Section 5.2.3 Communication”).

#### 5.2.3 Communication

Maybe the most important part of any scientist’s job is effective communication. Anyone can report sequence statistics, but it is the job of a scientist to distill data into information, take the information and communicate it as a coherent story, thereby creating knowledge about how the world works (Schimel, 2012). These stories are most often told via writing and speaking, reflecting the usual format of the exams and professional products of scientists (grant proposals, manuscripts, and lectures). However, communication can take other forms (videos, animations, infographics) and requires calibration to the level of formality of the medium (popular science magazine articles, general audience public radio interviews). In all cases, the scientist needs to convince the audience that their message is worth listening to, which requires both understanding the message they want to deliver (i.e., biological and bioinformatic literacy) and tailoring it to the interests and knowledge of an audience. For both quantifiable (accepted manuscripts, successful grant applications) and abstract (successfully making and maintaining collaborations, effective lectures) professional achievements, effective communication is the whole product (Hazelett, 2025). Delivering consistent clear messages requires frequent practice, with the best communicators contributing more to the global scientific enterprise.

## 6 Conclusion

Omics is a glue that connects biological fields–there are few biological questions that could not be enhanced with sequence-based analyses. Though sequencing is expensive, costs have plummeted, with the first human genome costing around $300 million (not-inflation adjusted) in 2001 (Service, 2006) to nearly $100 in 2024 (Liu et al., 2024). This cheaper sequencing has led databases to grow several million times larger in the last 20 years (Hug et al., 2025), increasing access. This access is supported by the development of tools that make data selection (Speth and Orphan, 2018; Maurya et al., 2022; Woodcroft et al., 2025) and use (Wright, 2024) less computationally demanding. Other groups have spent time creating integrated systems to simplify tool use (Anvi’o, Eren et al., 2015; QIIME2; Bolyen et al., 2019; mothur; Schloss, 2020). This combination of accessible data and tools has allowed unprecedented analyses using thousands of samples to identify new biomarkers of human health (Piccinno et al., 2025) and millions of samples to assess global patterns of microbiological distribution (Rodrigues et al., 2025). In this moment, sequence data generation will continue to be exponential, fueling a demand for scientists able to answer biological questions with increasingly large datasets (Stephens et al., 2015). Scientists able to understand and effectively find, analyze, and integrate this sequence data into larger biological narratives are poised to articulate biological processes from micron to global scales, an unprecedented opportunity. We believe this review provides a foundation for just such scientists.

BOX 1 Glossary of key terms

**rRNA**: The gene encoding, or the transcript that *is*, a part of the ribosome across all domains of life. The 16S rRNA gene and transcript are the most popular target for studying bacterial and archaeal evolution and diversity, though other rRNA subunits have been used and are informative for bacteria, archaea, and eukaryotes.
**Assembly**: The practice of reconstructing the sequences of the original nucleic acid molecules after their fragmentation before and during sequencing.
**Clone Library**: The product of inserting genetic material (targeted or untargeted) into a living vector (e.g., *Escherichia coli*), selecting for only the individuals that took up the material while separating genetically distinct clones (e.g., spread plating isolates with an antibiotic screen) allowing the vector to multiply the genetic material of interest sufficiently for further biochemical processing (e.g., sequencing).
**Contiguous Sequence (Contig)**: The shortest output from the assembly of nucleic acid reads (DNA or RNA). DNA contigs can be further refined into **scaffolds** and **chromosomes**.
**FAIR Data Practices**: A set of guidelines introduced by Wilkinson et al. (2016) outlining practices for ensuring long-term utility of published data, particularly though the promotion of consistent identifiers, rich metadata, and accessible file formats.
**Function Annotation**: The practice of assigning a sequence (often a predicted **ORF**) a function if it shows sufficient similarity to a sequence with known function (see **marker gene**).
**Genomics**: The study of life using the untargeted sequencing of DNA.
**Genome**: Strictly, the name for the complete set of **chromosomes** originating from the organism of study. Loosely, it also refers to assembled genomic material that is grouped into candidate genomes (**bins**, **MAGs**, and **SAGs**).
**[Finished, High, Medium, Low] Genome Quality**: Classifications of genome quality introduced by Bowers et al. (2017) relying on contiguity and estimates of completion and contamination.
**[Genomic] Bin**: A type of genome. A draft genome composed of contigs that have been grouped together based on similar characteristics (GC content, base frequency, or coverage), but have not yet been deemed of sufficiently high quality to be considered a usable genome/**MAG.**
**[Genomic] Chromosome**: A product of genomic sequence **assembly**, the product of combining **scaffolds** to produce a complete gap-free digitized representation of the source chromosome.
**[Genomic] Scaffolds**: A product of genomic sequence **assembly**, the product of combining multiple **contigs** with consistent orientation and defined gap sizes, though less complete than a **chromosome.**
**ISA Abstract Model**: Is used to organize metadata collection and distribution, and is a flexible organizing framework describing the metadata necessary to convey key elements of some data’s origin, including the Investigation, Study, and Assay that generated it.
**L50**: A metric for assessing an assembly’s quality via the number of assembled sequences. The metric of L50 describes how many of the longest sequences are needed to account for 50% of the assembly size. Assuming the assembly is of high quality and sufficient sequencing coverage, smaller L50 values indicate the assembly is composed of only a few sequences, which is considered a good thing.
**Machine Learning**: A class of tools developed from statistics (Bayesian statistics, game theory, computer vision) where algorithms are trained on existing data to identify patterns in new datasets leading to diverse kinds of artificial learning, including: reinforcement learning, supervised learning, and recently popular unsupervised generative learning (e.g., large language models).
**Metagenome-assembled Genome (MAG)**: A type of genome. A **bin** becomes a MAG after passing quality control standards (see **Genome Quality**).
**Marker Gene**: Genetic sequences that are strongly associated with a biological process of interest including but not limited to: phenotypes, evolution, or behavior.
**Minimum information checklist**: Is used to organize metadata collection and distribution and provides guidelines for required data reporting for data arising from specific classes of experiments or assays (see **ISA**) to ensure data usability without mandating exhaustive details.
**N50**: A metric for assessing an assembly’s quality via the length of contigs of an assembly, essentially a weighted median contig length. If all the contigs in an assembly are arranged from longest to shortest and began summing contig lengths one contig at a time, the N50 value would be the length of the contig where 50% of the total length has already been accounted for. Larger N50 values indicate that an assembly consists of longer contigs, generally indicating assembly success.
**[Meta]-Omics**: Omics is an analytical approach that studies entire sets of biological molecules (DNA, RNA, Proteins, Metabolites). Adding the prefix “meta” indicates that the analysis explicitly considers more than one organism (though non-meta omics may incidentally sequence more than one organism).
**Ontology**: A hierarchically structure for terminology where each term becomes increasingly specific while still “contained” within its broader term (e.g., a twig on a branch on a limb on a tree”) that is often used to describe gene functions (e.g., Gene Ontology) or metadata (e.g., The Environment Ontology).
**Open Reading Frame (ORF)**: An open reading frame is a predicted protein coding region from a nucleic acid sequence predicted due to the presence of genetic features characteristic of experimentally validated protein coding regions.
**Operational Taxonomic Unit (OTU)**: A label for sequences (reads to genomes) that have been deemed to share taxonomy based on **sequence clustering** at a defined percentage similarity threshold (often 95%).
**Sequence Clustering**: The practice of grouping like-with-like sequences (from reads to genomes), often using the measure of pairwise percentage similarity.
**Single amplified genome (SAG)**: A type of genome. A SAG is produced by sequencing the genomic material from a single cell, ensuring that the genomic environment is represented (distinct from a **MAG,** which may provide an incomplete understanding of the associated mobile genetic material or multiple chromosomes).
**Sequence Alignment**: The practice of comparing two sequences and searching for shared regions between the two. Often results in metrics describing the length of the aligning region and the percentage similarity.
**Sequence Library**: The name of the sequences originating from a single sample (e.g., a single metagenome file).
**Taxonomic Classification**: The practice of assigning a sequence a taxonomic origin based on its similarity to a reference sequence with assigned taxonomy.
**Transcriptomics**: The study of life using the untargeted sequencing of RNA.
